# Diptheria

**Published:** 1860-12

**Authors:** R. B. Ray

**Affiliations:** La Plata, Mo.


### ARTICLE 3.

### DIPHTHERIA.

BY R. B. RAY, M. D., LA PLATA, MO.

*To the Editors of the Chicago Medical Journal.*

Gentlemen:—As there has been an affection of the throat prevailing in this locality the past summer, it may be of some interest to your many readers, to give a brief sketch of a case which came under my care in the early part of September. As this case will give you a knowledge of this epidemic which has prevailed to so fearful extent, I will give some of the most prominent symptoms, diagnosis, arognosis and treatment, as well as my memory serves me. I was called Sunday, September 2d; I found patient, a boy, six years of age, breathing very laboriously, with full pulse, but moderately slow, some fever, surface hotter than normal. Upon an examination of the throat, I found the fauces much more red than natural, the uvula somewhat elongated, the tonsils swollen, as to those small white aphthous appearing specks seen in diphtheria, they were not well marked, but there was sufficient exudation to produce a stringy tenacious mass which is not unlike that found in pseudo-membraneous croup. I would remark here that the further development of the case exhibited a considerable quantity of yellowish pus tinged with blood; the cough was very troublesome, and was, of course, of hollow sound; dyspnœa, dysphagia, and aphnia were present; as to other symptoms, there were some present of minor import. As to the diagnosis, our practitioners differ much. Some call this disease ulcerated sore throat pseudo-membraneous croup, laryngitis, and so on. From what I have seen of those cases I shall be compelled to throw my opinion in favor of diphtheria. But without stopping to argue upon the diagnosis, I leave this part of my subject with my readers and pass to the treatment. The first I did was to give an emetic to free emesis, composed of ipecacuanha and sulph. zinc; embrocations to throat freely. The ipecac was continued just enough to nauseate and relax, while Dover's rondes, were given to procure rest. As a local application I applied tr. diluted muriatic acid, that is diluted with water half and half directly to the parts involved. This constituted the principal part of the treatment.

On the same day Dr. Ellis was called in consultation he endorsed the treatment already mentioned, therefore but little change was made. The next Monday my patient was worse, if possible, than the day previous. At this time all hope of recovery was lost, by ordinary medication, and explained fully to the parents, whereupon Dr. Still was summoned as additional council.

After consulting in the case and weighing the chances for and against tracheotomy, we came to the conclusion that there was no hope of recovery without the operation and but little with it, stating fully to the parents its dangers and the small chance of success, and that there was no chance of recovery without it. They chose the operation. It was performed by Dr. Still, assisted by Dr. Ellis and myself. The incision was made below the thyroid cartilage; the first incision through the skin brought into view and divided the superficial farcia; the thyroid gland was then put aside, when the cutting part of the operation was completed by severing the rings of the trachea. No blood was lost of any consequence; what was was readily controlled by hemo-statics; the canula was introduced by a little manipulation, and the operation bid fair to succeed up to 12 o'clock, Monday night, when the patient expired.

Directions were given to keep the canula open, but on removing it after death it was found closed up. Yet to my mind, this case, in all probability, would have been fatal.

---

